# Risk factors of metachronous brain metastasis in patients with EGFR-mutated advanced non-small cell lung cancer

**DOI:** 10.1186/s12885-020-07202-8

**Published:** 2020-07-28

**Authors:** Wen Ouyang, Jing Yu, Yan Zhou, Jing Hu, Zhao huang, Junhong Zhang, Conghua Xie

**Affiliations:** 1 Department of Radiation and Medical Oncology, Zhongnan Hospital of Wuhan University, 169 Donghu Road, Wuchang District, Wuhan, 430071 Hubei China; 2 Hubei Key Laboratory of Tumor Biological Behaviors, Zhongnan Hospital of Wuhan University, Wuhan, China; 3 Hubei Clinical Cancer Study Center, Zhongnan Hospital of Wuhan University, Wuhan, China

**Keywords:** Non-small cell lung cancer, Epidermal growth factor receptor, Brain metastases, Risk factor

## Abstract

**Background:**

NSCLC patients with EGFR mutation were at a higher incidence of developing brain metastasis (BM). Patients with BM are associated with high mortality. Reducing BM incidence becomes increasingly significant for NSCLC patients to achieve prolonged survival. The aim of the study was to explore the possible risk factors of developing metachronous BM during EGFR-TKIs treatment, and to identify the potential candidates for prophylactic cranial irradiation (PCI) or the first-line Osimertinib treatment.

**Methods:**

A total of 157 consecutive EGFR-mutated advanced NSCLC patients without BM at initial diagnosis in our institution from 2012 and 2018 were retrospectively reviewed. Comparisons of OS were performed based on BM status. The cumulative incidence of metachronous BM was calculated by the Kaplan-Meier method, and the independent risk factors of metachronous BM were investigated by multivariate analysis.

**Results:**

Patients developing metachronous BM had worse survival (mOS: 22.1 months) than patients not-developing BM (mOS: 44.8 months). Moreover, the multivariate analysis indicated that age ≤ 49 years (*P* = 0.035), number of extracranial metastases (*P* = 0.013), and malignant pleural effusion (*P* = 0.002) were independent risk factors of metachronous BM. Furthermore, the 1-year actuarial incidence of developing metachronous BM in patients with no risk factor (*n* = 101), 1 risk factor (*n* = 46), and 2 risk factors (*n* = 10) were 7.01, 14.61, and 43.75%, respectively (*P* < 0.001).

**Conclusions:**

Patients developing metachronous BM during EGFR-TKIs treatment have worse outcomes. Our results suggested that EGFR-mutated advanced NSCLC patients with ≥1 risk factors were candidates for PCI or the first-line Osimertinib treatment.

## Background

Lung cancer is the leading cause of cancer death all over the world [1]. Among them, 80–85% of patients are diagnosed as non-small cell lung cancer (NSCLC) [2]. Despite the presence of the blood-brain barrier (BBB), brain is still a frequent site of NSCLC metastasis. 10% of NSCLC patients present brain metastasis (BM) at their initial diagnosis, and 40–50% of patients develop metachronous BM during the course of the disease [3]. Patients with BM are associated with high mortality, poor prognosis, neurocognitive and life quality deficits [4]. Epidermal growth factor receptor tyrosine kinase inhibitors (EGFR-TKIs) largely improved the survival of EGFR-mutated advanced NSCLC patients [5–7]. Similarly, EGFR-mutated NSCLC patients with BM had a worse median OS of 25.1 months than the patients without BM (30.2 months) [8]. Whereas it was reported that NSCLC patients with EGFR mutation were at a higher incidence of developing BM than EGFR wild type [9–11]. Therefore, prevent the occurrence of metachronous BM becomes increasingly significant for EGFR-mutated advanced NSCLC patients to achieve prolonged survival.

How to reduce incidence of developing metachronous BM for EGFR-mutated advanced NSCLC patients? Firstly, prophylactic cranial irradiation (PCI) is a technique that delivers radiation therapy (RT) to the whole brain to prevent BM occurrence. It was reported to significantly reduce incidence of metachronous BM and improve overall survival (OS) in patients with limited-stage small cell lung cancer (SCLC) [12]. Whereas the results of RTOG-0214 on the effects of PCI in localized NSCLC patients indicated that PCI could reduce BM incidence, but failed to improve OS [13] and leaded to decline in immediate and delayed recall [14]. Interestingly, the 10-years update of RTOG-0214 showed that only patients non-operatively treated have an improved OS (*P* = 0.026, HR = 1.42, 95% CI: 1.04–1.94) and DFS (*P* = 0.014), implying that PCI might just benefit NSCLC patients with higher risk of BM. Secondly, Osimertinib is an oral, irreversible third-generation EGFR-TKIs with higher penetration in central nervous system (CNS) [15–17]. FLAURA study showed the lower frequency of CNS progression in the Osimertinib group than in the standard EGFR-TKIs group [17]. However, the first-line Osimertinib treatment for EGFR-mutated advanced NSCLC patients is not widely available in most developing countries due to its high cost. Therefore, it is higher cost-effective to apply the first-line Osimertinib treatment for EGFR-mutated NSCLC patients with higher risk of BM. These findings prompted us to identify population subsets with higher risk of BM, who are candidates for PCI or the first-line Osimertinib treatment.

Consequently, we established a retrospective single-institutional database including consecutive patients with EGFR-mutated advanced NSCLC between January 2012 and June 2018, to evaluate the impact of BM status on OS, to explore the possible risk factors for developing metachronous BM during the course of first-generation EGFR-TKIs therapy, and to identify the potential candidates for PCI or the first-line Osimertinib treatment.

## Methods

### Patients

**Fig. 1:**
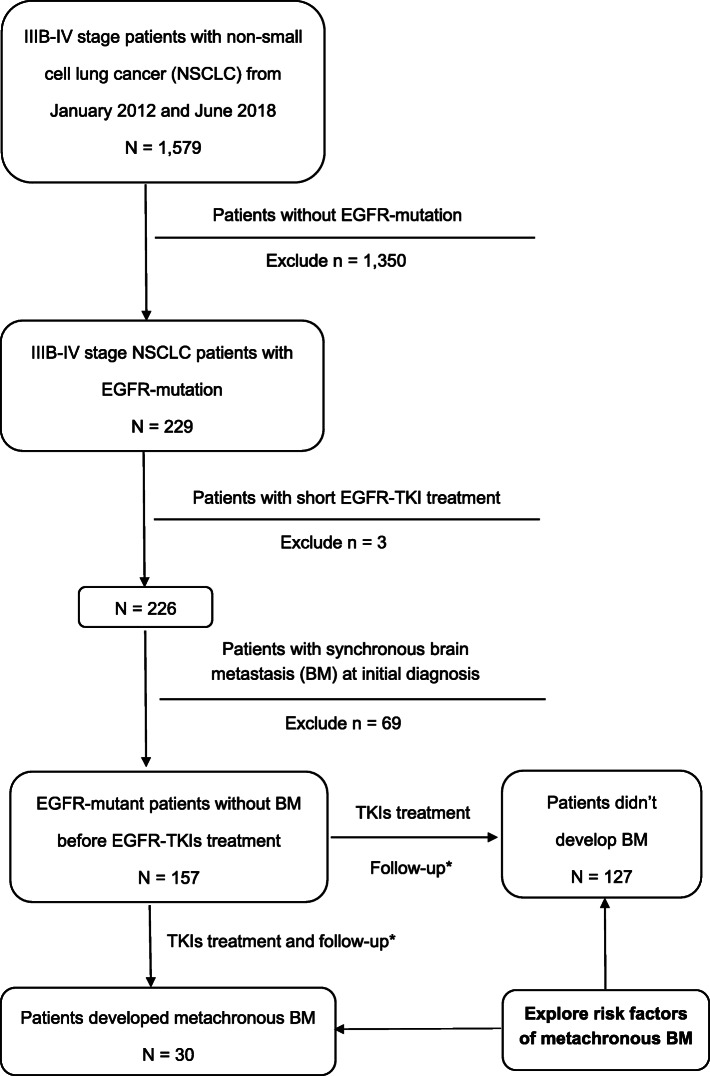
Flow chart for patients’ enrollment and treatment. *Follow-up examinations were performed every 2 months, including thoracic and abdominal CT scan, brain MRI scan

The flow chart of patient enrollment was shown in Fig. 1. Between January 2012 and June 2018, a total of 157 consecutive EGFR-mutated advanced NSCLC patients without BM at initial diagnosis were reviewed at the Department of Radiation and Medical Oncology, Zhongnan Hospital of Wuhan University. Our inclusion criteria are: (1) NSCLC was confirmed by cytology (14 pts), or histology (143 pts) (World Health Organization, WHO); (2) EGFR mutations were detected by real-time quantitative PCR (ARMS, 126 pts) or Next Generation Sequencing (NGS, 31 pts), using histological or cytological specimens from primary or metastatic lesions; (3) The disease was clinically diagnosed as stage IIIB (10 pts)-IV (147 pts) (American Joint Committee on Cancer, the 7th Edition); (4) Patients had negative results of enhanced magnetic resonance imaging (MRI, 149 pts) or computed tomography (CT, 8 pts) scans of brain before initial treatment; (5) Patients were treatment naive for EGFR-TKIs treatment. All patients received comprehensive assessments within 1 month before treatment, including physical and pathological examination, EGFR mutation test, and TNM stage evaluation.

### Treatment and follow up

Among the 157 patients without BM at initial diagnosis, 24 patients received chemotherapy as their first-line therapy, and the other 133 patients received EGFR-TKIs treatment initially. EGFR-TKIs (gefitinib, erlotinib, or icotinib) were continuously administered until progression of disease (PD) or intolerable side effects. Treatment beyond PD was allowed on the judgement of continuously clinical benefit by the oncologists.

Follow-up examinations were performed every 2 months, including thoracic and abdominal CT scan, brain MRI scan. Progression-free survival (PFS) was defined as the time from EGFR-TKIs treatment to PD (including local, regional, or distant progression) or death from any cause. OS was defined as the time from EGFR-TKIs treatment to death from any cause. Brain-metastasis-free survival (BMFS) was defined as the time from EGFR-TKIs treatment to BM occurrence. Treatment responses were evaluated by the response evaluation criteria in solid tumors as complete response (CR), partial response (PR), stable (SD), and progression (PD).

### Statistics

All statistical analyses were conducted using Statistical Package for Social Scientists (SPSS/Windows, Version 22.0, SPSS Inc., Chicago, USA). Descriptive statistics were used for categorical variables (frequency and percentage) and continuous variables (median and range). The cumulative incidence of BM and survival were calculated by the Kaplan-Meier method with 95% confidence intervals (CIs). Univariable and multivariable Cox regression analyses were performed to explore the risk factors of metachronous BM. The multivariable Cox regression analysis simultaneously included factors that had shown associations (*P* < 0.100) in the univariable Cox regression analyses, and variables based on their clinical significance according to previously literature reports. The optimal cut-off values of continuous valuables were calculated by X-tile software [18]. All tests were two-sided and *P* < 0.05 were considered statistically significant.

## Results

### Patient characteristics

The flow chart of patient enrollment was shown in Fig. 1. Between January 2012 and June 2018, Among the 229 consecutive patients with EGFR-mutated advanced NSCLC, three patients were excluded due to short EGFR-TKI treatment (< 1 month), and 69 patients were excluded due to synchronous BM. A total of 157 patients without BM at initial diagnosis were included: 30 patients (19.1%) developed metachronous BM during EGFR-TKIs treatment and 127 patients (80.9%) didn’t. Among the 30 patients with metachronous BM, 20 patients (20/30, 66.7%) first progressed in intracranial disease, implying metachronous BM principally correlated with the ability of EGFR-TKIs to pass through BBB.

The clinical and treatment characteristics of these patients grouped by BM status are shown in Table 1. The median age of the patients without BM and patients developing metachronous BM was 60 and 54 years, respectively. Patients who would develop metachronous BM were more likely to have a more favorable Karnofsky Performance Status (KPS score ≥ 80: 100% patients developing metachronous BM vs. 90.5% patients without BM). There was no difference between the two groups with respect to gender, histology, BMI, smoking status, tumor markers level, clinical stages, and extracranial metastatic location. In addition, it was reported that the type of EGFR mutations and were associated with OS, whereas there was no difference on the proportion of EGFR mutations type between the two groups grouped by BM status (χ2 = 3.084, *P* = 0.214), indicating the similar distribution of EGFR mutations type had no significant impact on OS between the two groups grouped by BM status.

**Table 1:** Baseline and treatment characteristics of patients grouped by BM status

	Patients without BM (*n* = 127)		Patients developing metachronous BM (*n* = 30)	
Characteristic	NO.	%	NO.	%
Age, years				
≤ 49	24	18.9	12	40.0
> 49	103	81.1	18	60.0
Median (Range)	60 (28–93)		54 (33–75)	
Gender				
Male	63	49.6	14	46.7
Female	64	50.4	16	53.3
KPS score				
≥ 80	115	90.5	30	100
< 80	12	9.5	0	0
Histology				
Adenocarcinoma	122	96.1	28	93.3
Non-adenocarcinoma	5	3.9	2	6.7
BMI				
Mean (95%CI)	21.9 (14.9–28.8)		22.7 (16.3–29.2)	
Smoking status				
Yes	42	33.1	8	26.7
No	85	66.9	22	73.3
CEA (ng/ml)				
Median (Range)	23.5 (0.5–8048)		30.5 (1.5–1819)	
CA125 (ng/ml)				
Median (Range)	52.9 (4.76–3369)		69.4 (11.3–954.5)	
NSE (ng/ml)				
Median (Range)	15.0 (4.4–133.1)		15.2(7.6–55.2)	
First-line treatment regimen				
EGFR-TKI treatment	112	88.2	21	70
Chemotherapy	15	11.8	9	30
Type of EGFR mutations				
L858R	49	38.6	14	46.7
19 deletion	67	52.8	11	36.7
Other^a^	11	8.6	5	16.7
NO. of extracranial metastases				
0	8	6.3	2	6.7
1	65	51.2	14	46.7
2	42	33.1	10	33.3
3 or more	12	9.4	4	13.3
Clinical stages				
Stage IIIB	8	6.3	2	6.7
Stage IV	119	93.7	28	93.3
Location of extracranial metastatic sites				
Pleural effusion	8	6.3	6	20.0
Liver	17	13.4	4	13.3
Adrenal	17	13.4	1	3.3
Bone	73	57.5	18	60
Lung	75	59.1	17	56.6
Other	12	9.4	2	6.7
Types of EGFR-TKIs				
Gefitinib	80	63.0	19	63.3
Erlotinib	31	24.4	7	23.3
Icotinib	16	12.6	4	13.4
Local therapy for BM				
None	NA		8	26.7
WBRT	NA		14	46.7
SRS	NA		8	26.6

*Abbreviation*: *BM* brain metastasis, *KPS* Karnofsky Performance Status, *CI* confidence interval, *EGFR* epidermal growth factor receptor, *WBRT* whole brain radiation therapy, *SRS* stereotactic radiosurgery, *NSE* neuron-specific enolase, *TKI* tyrosine kinase inhibitor

^a^Uncommon EGFR mutations, including 20-ins (7 pts), G719X (3 pts), L816Q (2 pts), G863D (1 pt), K846R (1 pt), V765A (2 pts)

### The incidence of metachronous BM and survival

**Fig. 2:**
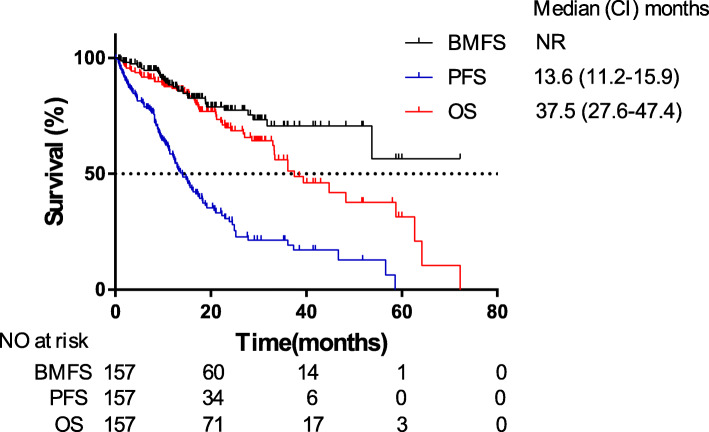
Kaplan-Meier plot of OS, PFS, and BMFS in EGFR-mutated advanced NSCLC patients without BM at initial diagnosis. OS, overall survival; PFS, progression-free survival; BMFS, brain-metastasis-free survival; NSCLC, non-small cell lung cancer; BM, brain-metastases; CI, confidence interval

The median duration of follow-up was 24.1 months (95% CI: 19.6–28.6 months). Thirty patients (19.1%) developed metachronous BM during EGFR-TKIs treatment. Among them, patients with symptomatic and asymptomatic BM were 18 (60%) and 12 (40%) respectively. Fourteen patients (46.7%) received WBRT and 8 patients (26.7%) received stereotactic radiosurgery (SRS) plus continuous EGFR-TKIs treatment, other 2 patients (6.7%) received continuous EGFR-TKIs plus supportive care, and 6 patients (20%) switched to chemotherapy. In addition, 9 patients (9/30, 30%) receiving chemotherapy as the first-line treatment developed metachronous BM during subsequent EGFR-TKIs therapy. The 1-, 2- and 3-year risks of BM were 11.6, 22.6 and 29.4% respectively (Fig. 2).

The median OS of these 157 patients was 37.5 months (95% CI: 27.6–47.4 months). The 1-, 2- and 3-year OS rates were 86.9, 69.8 and 55.9% respectively (Fig. 2). For PFS, 105 patients (66.9%) progressed during follow-up time. Among them, a total of 51 patients (38 of patients without BM group [38/127, 29.9%] and 13 of patients developing metachronous BM group [13/30, 43.4%]) finally received Osimertinib treatment after the detection of T790M mutation indicated positive by plasma or tissue rebiopsy specimens. Median PFS was 13.6 months (95% CI:11.2–15.9 months). The 1-, 2- and 3-year PFS rates were 57.8, 29.4 and 21.3% respectively (Fig. 2). Our median OS and PFS were longer than those of the clinical trials for patients with EGFR-mutated advanced NSCLC [19].

The overall response rates were partial for 76.4%, stable for 23.0%, and progressive for 0.6% of EGFR-TKIs treatment at the first follow-up examination.

### Overall survival of patients grouped by BM status

**Fig. 3:**
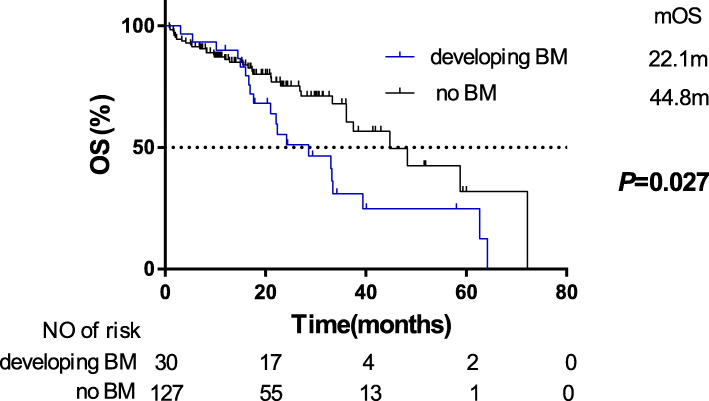
Kaplan-Meier plot of OS in patients with EGFR-mutated advanced NSCLC grouped on BM status. OS, overall survival; NSCLC, non-small cell lung cancer; BM, brain-metastases; CI, confidence interval

To evaluate the impact of BM status on OS, the 157 patients were grouped by with metachronous BM and without BM. Compared with patients without BM, patients developing metachronous BM during the course of EGFR-TKIs treatment were at a higher risk on OS (HR = 1.86, 95%CI:1.07–3.26). Our findings confirmed that patients developing metachronous BM during EGFR-TKIs treatment had poorer outcomes (median OS: 22.1 months) than patients without BM (median OS: 44.8 months, Fig. 3).

### Risk factors of developing metachronous BM

Several clinical factors were associated with metachronous BM in both univariate and multivariate analyses (Table 2). In univariate analyses, BM was associated with age, the first-line treatment regimens, types of EGFR mutations, numbers of extracranial metastases, and malignant pleural effusion. Other factors such as gender, KPS score, smoking status, tumor marker levels before treatment, clinical stages, types of EGFR-TKIs, and metastatic locations were not associated with metachronous BM.

**Table 2:** Univariate and multivariate analyses for the factors associated with risks for metachronous BM

Factors	Univariate analysis Incidence of BM (%)			Multivariate analysis Incidence of BM (%)		
	HR	95%CI	*P*	HR	95%CI	*P*
Gender: female VS male	1.139	0.556–2.337	0.772	1.495	0,506–4.421	0.467
Age, years	0.963	0.931–0.995	0.023			
> 49 VS ≤ 49	0.341	0.162–0.720	0.005	0.396	0.167–0.938	0.035
KPS score: < 80 VS ≥80	0.045	0.000–40.173	0.371			
BMI	1.035	0.922–1.161	0.562	1.057	0.919–1.216	0.436
Smoking: yes VS no	0.798	0.353–1.801	0.586	1.302	0.384–4.408	0.672
Tumor markers level before treatment						
CEA (ng/ml)	1.000	0.999–1.000	0.685	1.000	0.999–1.000	0.294
CA125 (ng/ml)	1.000	0.998–1.001	0.498			
NSE (ng/ml)	1.014	0.985–1.043	0.351			
First-line treatment regimen						
Chemotherapy VS EGFR-TKI	2.296	1.050–5.018	0.037	0.504	0.153–1.660	0.260
Type of EGFR mutations			0.071			0.061
19-del VS L858R	0.579	0.263–1.277	0.176	0.490	0.201–1.194	0.116
Other^a^ VS L858R	1.968	0.703–5.508	1.968	2.408	0.566–10.246	0.234
Clinical stages: IIIB VS. IV	0.501	0.152–1.653	0.257			
Type of EGFR-TKIs			0.262			
Erlotinib VS Gefitinib	0.422	0.118–1.503	0.183			
Icotinib VS Gefitinib	0.460	0.109–1.946	0.292			
NO. of extracranial metastasis						
0–2 VS 3 or more	0.523	0.181–1.514	0.232	0.200	0.056–0.713	0.013
Location of extracranial metastasis						
Pleural effusion	3.245	1.300–8.098	0.012	5.283	1.854–15.053	0.002
Liver	1.066	0.371–3.062	0.906			
Adrenal	0.242	0.033–1.779	0.163			
Bone	1.161	0.558–2.413	0.690			
Lung	1.543	0.685–3.475	0.295			
Other	1.332	0.317–5.605	0.696			

*Abbreviation*: *BM* brain metastasis, *KPS* Karnofsky Performance Status, *CI* confidence interval, *EGFR* epidermal growth factor receptor, *WBRT* whole brain radiation therapy, *SRS* stereotactic radiosurgery, *NSE* neuron-specific enolase, *TKI* tyrosine kinase inhibitor

^a^Uncommon EGFR mutations, including 20-ins (7 pts), G719X (3 pts), L816Q (2 pts), G863D (1 pt), K846R (1 pt), V765A (2 pts)

The factors showing associations (*P* < 0.100) in the univariable Cox regression analyses, as well as other factors that were reported to be associated with BM in previous studies [20, 21] were further examined by multivariable Cox regression analysis. Results of multivariate analysis indicated that age ≤ 49 years (*P* = 0.035), numbers of extracranial metastases (*P* = 0.013), and documented malignant pleural effusion (*P* = 0.002) were independent high-risk factors of developing metachronous BM, while the first-line treatment regimens and types of EGFR mutations were not associated with metachronous BM in multivariate Cox regression analysis.

**Fig. 4:**
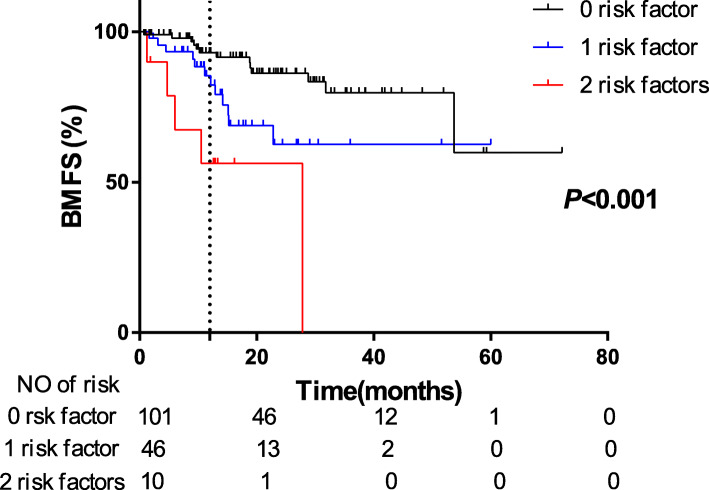
Comparison of the actuarial risk of developing metachronous BM among patients with different numbers of risk factors

Furthermore, the 1-year actuarial incidence of developing metachronous BM in patients with no risk factor (*n* = 101), 1 risk factor (*n* = 46), and 2 risk factors (*n* = 10) were 7.01, 14.61, and 43.75%, respectively (*P* < 0.001, Fig. 4). Meanwhile, we performed an internal validation by randomly selecting 52 cases from our patient cohort. The 1-year actuarial incidence of developing metachronous BM in these 52 patients with no risk factor (*n* = 37), 1 risk factor (*n* = 11), and 2 risk factors (*n* = 4) were 5.65, 22.2, and 50.0%, respectively (*P* = 0.004, Figure S1). These results were consistent with the whole cohort, indicating the credibility of the result to some extent. Therefore, patients with more risk factors had higher risk of developing metachronous BM. Our studies suggested that the patients with ≥1 risk factors were more likely to benefit from PCI or the first-line Osimertinib treatment.

## Discussion

EGFR mutations are observed in approximately 10–15% of the Caucasian population [22] and more than 50% of the Asian population [23] with non-squamous NSCLC. During the past two decades, the advances of EGFR-TKIs revolutionarily improved the prognosis of patients with EGFR-mutated advanced NSCLC. The WJTOG3405 trial reported that the median OS of EGFR-mutated advanced NSCLC patients treated with first-generation EGFR-TKI was up to 30.2 months [24]. Our results of 157 EGFR-mutated advanced NSCLC patients without BM at initial diagnosis also showed a median OS of 37.5 months (Fig. 2). Compared with chemotherapy, although EGFR-TKIs were reported to pass through BBB and reduce BM among EGFR-mutated NSCLC patients [25, 26], there remain some patients developing metachronous BM during the course of EGFR-TKIs therapy. Lee et al. found that 26% of the patients developed central nervous system (CNS) failure and 13% experienced isolated CNS failure among 166 patients with a clinical benefit to first-generation EGFR-TKIs (gefitinib or erlotinib) treatment [27]. In our study, 30 patients (30/157, 19.1%) developed metachronous BM during first-generation EGFR-TKIs treatment, and 1-, 2- and 3-year risks of developing BM were 11.6, 22.6 and 29.4% respectively (Fig. 2). Moreover, patients with longer survival exposed to a higher risk of BM [9]. Therefore, the first-generation EGFR-TKIs therapy resulted in decreased risk of non-BM lesions but had limited impact on BM.

It was well known that BM is a common reason leading to treatment failure [28]. In our study, compared with patients without BM, patients developing metachronous BM during the course of first-generation EGFR-TKIs treatment were at a higher risk on OS (HR = 1.86, 95%CI:1.07–3.26) (Fig. 3), which was on the condition that there was no difference on clinical and treatment characteristics between the two groups grouped by BM status (Table 1). Among these clinical and treatment characteristics, it was reported that the type of EGFR mutation was associated with OS. And the median OS of our patients with L858R, 19-del, and uncommon EGFR mutations was 38.1 months, 45.1 months, and 24.1 months, respectively (*P* = 0.026). However, there was no difference on the proportion of EGFR mutation type between the two groups grouped by BM status (χ2 = 3.084, *P* = 0.214), indicating the similar distribution of EGFR mutation type had no significant impact on OS between the two groups. Therefore, reducing incidence of BM in EGFR-mutated advanced NSCLC patients becomes increasingly significant to achieve prolonged survival.

The use of PCI or the first-line Osimertinib treatment could reduce incidence of metachronous BM among EGFR-mutated advanced NSCLC patients. However, existing evidences suggest that PCI might just suitable for patients with high risk of developing BM, and the high cost of Osimertinib leaded to the limitation of first-line Osimertinib treatment in most developing countries. Therefore, it is important to identify population subsets with higher risk of BM as candidates for PCI or the first-line Osimertinib treatment. Previous studies identified several risk factors of BM in NSCLC, including younger age [29–31], non-squamous cell carcinoma [29], high serum CEA level [20], and disease stages [30, 32]. However, they were not specific for EGFR-mutated advanced NSCLC patients, and synchronous BM at initial diagnosis and metachronous BM during their disease course are seldom differentiated in these reports.

In our current study, multivariate analysis indicated that age ≤ 49 years was correlated with higher risk of metachronous BM (Table. 2). Despite the difference of age cut-off, our results were consistent with previous studies [30, 33]. The underlying mechanism remains unclear. It was partly interpreted that young people may have better performance status, which is associated with longer survival, leading to higher risk of exposure to BM. Moreover, several studies have shown that BM is associated with the angiogenic microenvironment, and the cerebrovascular microenvironment factors of young patients may be better than those of older patients [34]. Further investigations are required to identify the specific mechanism that younger patients are more likely to develop BM.

The numbers of malignant pleural effusion and extracranial metastases were also independent risk factors of metachronous BM (Table. 2). The underlying mechanism was also unclear. It may be interpreted that both pleural effusion and BM is associated with the angiogenic microenvironment [34]. In addition, the numbers of extracranial metastases are reflection of tumor burden, which was positive correlated with the development of BM.

Furthermore, our results confirmed that the predictive value of gender and KPS score for metachronous BM may remain controversial [35]. Previous studies reported that elevated CEA [20, 21, 35], NSE [29], and CA125 [29] were independent risk factors of BM. However, there is no correlation between tumor markers levels before treatment (including CEA, NSE, and CA125) and the metachronous BM in our study. And the first-line treatment regimen was also not associated with metachronous BM in our multivariate Cox analysis. In addition, a recent retrospective study [21] also showed that point mutations in exon 21 were independent risk factors of BM. However, our results failed to show a statistical difference in the association between types of EGFR mutations and metachronous BM.

Finally, the 1-year actuarial risk of developing metachronous BM in patients with no risk factor (*n* = 101), 1 risk factor (*n* = 46), and 2 risk factors (*n* = 10) were 7.01, 14.61, and 43.75%, respectively (*P* < 0.001, Fig. 4). Obviously, patients with more risk factors had higher risk of developing metachronous BM. Our studies suggested that the patients with ≥1 risk factors were more likely to benefit from PCI or were candidates for the first-line Osimertinib treatment. Certainly, there are several limitations in our study, this was a retrospective study in a single institution, which inevitably resulted in a selection bias. More finely devised prospective and random study is needed to confirm our results, and the mechanisms of the correlation between these risk factors and metachronous BM are to be further explored.

## Conclusions

Collectively, the findings of this study were as follows. First, our study confirmed EGFR-mutated advanced NSCLC patients with metachronous BM had worse outcomes. Second, the multivariate Cox analysis indicated that younger age (≤ 49 years), more extracranial metastases, and malignant pleural effusion were independent risk factors of metachronous BM. Third, the patients with more risk factors were more likely to benefit from PCI or the first-line Osimertinib treatment.

## Supplementary information

**Additional file 1: Figure S1.** Comparison of the actuarial risk of developing metachronous BM among randomly select 52 cases from our patient cohort grouped by different numbers of risk factors.

## Data Availability

The datasets used and analyzed in the current study are available from the corresponding author upon reasonable request.

## Abbreviations

BM: Brain metastasis
PCI: Prophylactic cranial irradiation
BBB: Blood-brain barrier
CI: Confidence interval
EGFR: Epidermal growth factor receptor
NSCLC: Non-small cell lung cancer
SCLC: Small cell lung cancer
WBRT: Whole brain radiation therapy
SRS: Stereotactic radiosurgery
CNS: Central nervous system
NGS: Next Generation Sequencing
NSE: Neuron-specific enolase
DFS: Disease free survival
OS: Overall survival
PD: Progression of disease
PFS: Progression-free survival
BMFS: Brain-metastasis-free survival
CR: Complete response
PR: Partial response
SD: Stable
TKI: Tyrosine kinase inhibitor
VEGF: Vascular endothelial growth factor
