# Supplementary figures and images for: Risk factors of metachronous brain metastasis in patients with EGFR-mutated advanced non-small cell lung cancer
Source: BMC Cancer. 2020 Jul 28;20:699. doi: 10.1186/s12885-020-07202-8 (PMC7390194; doi:10.1186/s12885-020-07202-8)

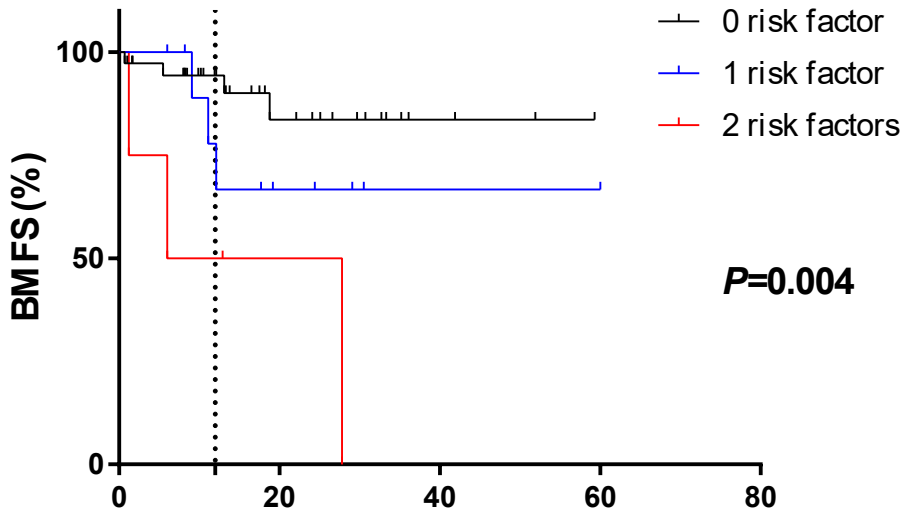

| NO of risk     |    | Time(months) |   |   |   |
|----------------|----|--------------|---|---|---|
| 0 risk factor  | 37 | 13           | 3 | 0 | 0 |
| 1 risk factor  | 11 | 4            | 1 | 0 | 0 |
| 2 risk factors | 4  | 1            | 0 | 0 | 0 |

Supplement: Supplementary file 1 — Additional file 1: Figure S1. Comparison of the actuarial risk of developing metachronous BM among randomly select 52 cases from our patient cohort grouped by different numbers of risk factors. [file 12885_2020_7202_MOESM1_ESM.pdf]
